# The Aachen Falls Prevention Scale: Multi-Study Evaluation and Comparison

**DOI:** 10.2196/12114

**Published:** 2019-05-16

**Authors:** Peter Rasche, Verena Nitsch, Lars Rentemeister, Mark Coburn, Benjamin Buecking, Christopher Bliemel, Leo Cornelius Bollheimer, Hans-Christoph Pape, Matthias Knobe

**Affiliations:** 1 Institute of Industrial Engineering and Ergonomics Department of Mechanical Engineering RWTH Aachen University Aachen Germany; 2 Department of Orthopaedic Trauma University of Aachen Medical Center RWTH Aachen University Aachen Germany; 3 Klinik für Anästhesiologie University of Aachen Medical Center RWTH Aachen University Aachen Germany; 4 Center for Orthopaedics and Trauma Surgery University Hospital of Giessen and Marburg Marburg Germany; 5 Department of Geriatrics University of Aachen Medical Center RWTH Aachen University Aachen Germany; 6 Department of Orthopaedic Trauma University of Zurich Medical Center University of Zurich Zurich Switzerland

**Keywords:** meta-analysis, elderly, self-assessment, hip injuries, leg injuries, sensitivity, specificity

## Abstract

**Background:**

Fall risk assessment is a time-consuming and resource-intensive activity. Patient-driven self-assessment as a preventive measure might be a solution to reduce the number of patients undergoing a full clinical fall risk assessment.

**Objective:**

The aim of this study was (1) to analyze test accuracy of the Aachen Falls Prevention Scale (AFPS) and (2) to compare these results with established fall risk assessment measures identified by a review of systematic reviews.

**Methods:**

Sensitivity, specificity, and receiver operating curves (ROC) of the AFPS were calculated based on data retrieved from 2 independent studies using the AFPS. Comparison with established fall risk assessment measures was made by conducting a review of systematic reviews and corresponding meta-analysis. Electronic databases PubMed, Web of Science, and EMBASE were searched for systematic reviews and meta-analyses that reviewed fall risk assessment measures between the years 2000 and 2018. The review of systematic reviews was conducted in accordance with the Preferred Reporting Items for Systematic Reviews and Meta-Analysis statement. The Revised Assessment of Multiple SysTemAtic Reviews (R-AMSTAR) was used to assess the methodological quality of reviews. Sensitivity, specificity, and ROC were extracted from each review and compared with the calculated values of the AFPS.

**Results:**

Sensitivity, specificity, and ROC of the AFPS were evaluated based on 2 studies including a total of 259 older adults. Regarding the primary outcome of the AFPS *subjective risk of falling*, pooled sensitivity is 57.0% (95% CI 0.467-0.669) and specificity is 76.7% (95% CI 0.694-0.831). If 1 out of the 3 subscales of the AFPS is used to predict a fall risk, pooled sensitivity could be increased up to 90.0% (95% CI 0.824-0.951), whereas mean specificity thereby decreases to 50.0% (95% CI 0.42-0.58). A systematic review for fall risk assessment measures produced 1478 articles during the study period, with 771 coming from PubMed, 530 from Web of Science, and 177 from EMBASE. After eliminating doublets and assessing full text, 8 reviews met the inclusion criteria. All were of sufficient methodological quality (R-AMSTAR score ≥22). A total number of 9 functional or multifactorial fall risk assessment measures were extracted from identified reviews, including Timed Up and Go test, Berg Balance Scale, Performance-Oriented Mobility Assessment, St Thomas’s Risk Assessment Tool in Falling Elderly, and Hendrich II Fall Risk Model. Comparison of these measures with pooled sensitivity and specificity of the AFPS revealed a sufficient quality of the AFPS in terms of a patient-driven self-assessment tool.

**Conclusions:**

It could be shown that the AFPS reaches a test accuracy comparable with that of the established methods in this initial investigation. However, it offers the advantage that the users can perform the self-assessment independently at home without involving trained health care professionals.

## Introduction

### Background

Fall incidents are an increasing problem in aging societies [1]. Every third adult older than 65 years falls at least once a year [2]. Increased morbidity and mortality are typical consequences of this fall incidence or related injuries [3-5]. In addition, each individual’s risk of falling is generally difficult to detect and is likely to be underestimated [2]. Thus, routinely assessing an individual’s fall risk is recommended within the United States, United Kingdom, and Germany [6-8]. This assessment is mainly carried out by the family doctor and is based on the question about fall incidents or the subjective fear of falling. In the event that a potential risk is identified, further functional or multifactorial case risk assessment measures are applied. The National Institute for Health and Care Excellence (NICE, United Kingdom) recommends a multifactorial assessment for suspected cases of outpatients, which aims at different risk factors and does not only evaluate the pure mobility of the patients [7]. The United States Preventive Service Task Force (US PSTF, United States), on the other hand, recommends keeping the assessment as simple as possible and asking patients about their fall history as well as carrying out a functional assessment such as the Timed Up and Go Test [8]. The German College of General Practitioners and Family Physicians recommends a similar assessment as the US PSTF based on questioning patients’ fall history and assessing their fall risk based on a functional test like the Timed Up and Go test [6]. A comparison of these guidelines shows that there is still no common best practice for assessing individual fall risks in different industrial nations. What all 3 approaches have in common, however, is that the initiative to carry out an assessment always emanates from the treating family doctor and, in addition, because of a multifactorial or functional assessment, is very resource-intensive and time-consuming [9-13]. Many of them are also problematic in terms of their interrater reliability [14-19]. Preventive measures are, thus, difficult and demand sufficient integration and implementation into aftercare and outpatient management [9,10,14,20,21]. Especially if patients’ fall risk should be monitored over a long term, clinical assessment measures are oversized and unsuitable, particularly in terms of a low-threshold service. This leads to the inclusion of a high proportion of low-risk people and waste of resources in terms of clinical setting.

Patient-driven self-assessment as a preventive measure might be a solution [14,22,23]. A promising approach for patient-guided self-assessment of personal fall risk is the Aachen Falls Prevention Scale (AFPS) [11]. On the basis of a 3-step multifactorial and functional assessment, users can evaluate their personal risk of falling. The first step includes 10 yes/no questions (subscale 1) covering typical risk factors such as cognitive or visual impairment, continence problems, falls history, footwear that is unsuitable, health problems that may increase their risk of falling (osteoporosis, Parkinson, arthrosis, or rheumatic disease), or medication. The second step involves a 10-second free-standing test (subscale 2), enabling the users to identify certain balance problems by themselves. The third step is a self-evaluation on a 10-point Likert-type scale (third subscale and primary outcome of the AFPS). Users should rate their subjective risk of falling in regard to the results of the risk factor assessment and the balance test [11]. Thus, the AFPS incorporates a multifactorial fall risk assessment as recommended by NICE or the US PSTF [7,8]. The scale is designed to be used by older adults themselves. This is a benefit compared with most multifactorial fall risk assessments, as is mentioned before. In addition, older adults could perform this self-assessment on their own using a paper version of the AFPS or the corresponding Aachen Falls Prevention App (AFPA) [24]. Thus, older adults get empowered to assess their risk of falling on their own and to consult a physician in advance. In addition, it is also possible to reach groups of people who do not regularly take part in preventive examinations or checkups with their family doctor. For example, the corresponding self-assessment can be sent by the health insurance company. The advantage for the physicians treating the patients is that the self-assessment of the patient provides them with direct information about the existence of risk indicators. In addition, regular use of the AFPS will give an overview of these risk indicators, of balance problems, or of the self-perceived risk of falls changing over time. However, it is still open to what extent the AFPS is covered by clinical multifactorial assessments, which are carried out in a clinical environment with a high time and personnel expense.

### Aim of This Study

The purpose of this study was (1) to analyze the test accuracy of the AFPS and (2) to compare these results with established fall risk assessment measures identified by a review of systematic reviews.

## Methods

The objective of this study was to determine the sensitivity, specificity, and area under the curve (AUC) via receiver operating curves (ROCs) of the AFPS and to compare them with established measures. Accordingly, the methodological approach of this work is divided into 2 steps. In the first step, the corresponding characteristic values (sensitivity, specificity, and AUC) are calculated. In the second step, a systematic literature search was carried out with the aim of identifying relevant reviews of established case risk assessment measures so that the calculated AFPS characteristics can be compared with these.

### Test Accuracy Analysis of the Aachen Falls Prevention Scale

The first objective of this study was to determine the test accuracy of the AFPS. The AFPS has 3 binary outcomes (positive/negative) associated with the 3 steps performed during self-assessment. Sensitivity and specificity were calculated regarding the primary outcome of the AFPS *subjective risk of falling*. Subsequently, sensitivity and specificity were calculated in the case that 1 out of the 3 outcomes of the AFPS identified a positive overall result. The same procedure was applied for the calculation of the ROC and thus the AUC values.

#### Sensitivity and Specificity

Sensitivity and specificity were calculated as described by Lalkhen and McCluskey, Lusardi et al, and Oliver et al [25-27]. Calculations were performed using Statistical Package of Social Science (SPSS) version 22 (IBM Corp). Pooled sensitivity and specificity were calculated using Meta-DiSc1.4 (Clinical Biostatistics team of the Ramón y Cajal Hospital in Madrid) [28].

#### Receiver Operating Curves and Area Under the Curve

Second, the reliability of the AFPS was analyzed by plotting ROCs. These curves plot the *sensitivity* against (*1-specificity*) for all possible parameter values. The ROC and the line of no discrimination (diagonal) differ from each other if the parameters analyzed are not randomly related. The AUC could be used to quantify this result. In case of a random relationship, the AUC value is 0.5. In the range between 0.5 and 0.7 for the AUC value, a test is considered less accurate, whereas in the range from greater than 0.7 to 0.9, it is considered moderately accurate. A perfect test would have an AUC value of 1 [29,30]. ROCs and AUCs were calculated using Bland-Altman analyses performed in SPSS separately for both studies.

#### Data Collection

Data from 2 studies by Knobe et al and Rasche et al, in which the AFPS was included, were used for test accuracy analysis [31,32]. In both studies, identification of fallers was performed according to the definition by Panzer et al [12,31,32]. Utilizing the fall risk screening criteria, participants reporting greater than or equal to 2 noninjury falls in the past year or greater than or equal to 1 injury fall were categorized as *fallers*; participants reporting no falls were categorized as *nonfallers* [31]. The test accuracy of the AFPS was calculated compared with this binary classification.

### Comparison of the Aachen Falls Prevention Scale With Established Assessment Measures

The second aim of this study was to compare the sensitivity, specificity, and AUC values of the AFPS with established fall risk assessment measures. Hence, a review of systematic reviews was conducted to retrieve reliable sensitivity, specificity, and AUC values from literature. This review of systematic reviews was carried out in accordance with the Preferred Reporting Items for Systematic Reviews and Meta-Analysis (PRISMA) statement [33].

#### Inclusion and Exclusion Criteria

Only reviews that fulfilled the following criteria were included: (1) published between the years 2000 and 2018, (2) stating specific values for sensitivity and specificity or AUC, (3) including fall risk assessment measures designed for outpatient application, (4) no specific investigation of a diseased subgroup of older adults, such as, for example, dementia patients.

#### Search Methods

Due to the aim of this study, only the electronic databases PubMed, EMBASE, and Web of Science were searched in July and November 2018. The search term was, because of the purpose of this study, defined as *fall risk assessment*. Reference lists from the identified publications were reviewed to identify additional research articles of interest.

#### Selection Process

Titles of records resulting from the literature search were independently screened by the first author and discussed with the coauthors. When further clarification was needed, the abstracts were consulted, and in a third step, the full text was retrieved. Disagreements were resolved by the senior author.

#### Data Extraction and Management

The authors extracted the following data and resolved any disagreements in consultation with the senior author: (1) authorship and publication-related information; (2) name of fall risk assessment measures reviewed; (3) overall sample size; (4) sensitivity and specificity values of the fall risk assessment measures; (5) and if available, AUC value for the fall risk assessment measures. Data were only extracted for the case risk assessment measures, which were examined in at least 2 of the 8 identified reviews. This procedure should ensure that the comparison was not based solely on the data from a single review. Furthermore, this procedure should ensure that scientifically relevant and correspondingly frequently discussed fall risk assessment measures were included in the comparison.

#### Methodological Quality Assessment

The Revised Assessment of Multiple SysTemAtic Reviews (R-AMSTAR) was used to quantitatively evaluate the methodological quality of identified systematic reviews regarding the inclusion in this study [34]. Reviews are evaluated by the presence of the following 11 domains: (1) an a priori design, (2) duplicate study selection and data extraction, (3) a comprehensive literature search, (4) the use of status of publication as an inclusion criteria, (5) a list of included/excluded studies, (6) characteristics of included studies, (7) documented assessment of the scientific quality of included studies, (8) appropriate use of the scientific quality in forming conclusions, (9) the appropriate use of methods to combine findings of studies, (10) assessment of the likelihood of publication bias, and (11) documentation of conflicts of interest [35]. Each domain is rated on a 4-point scale, whereas R-AMSTAR total scores range from 11 to 44 points. For inclusion of the evaluated review, a total score of 22 points was required [34]. The authors in charge of extracting data from the selected reviews also preliminarily and independently assessed the methodological quality of the contributions. The supervising author resolved any discrepancies.

#### Data Synthesis

Identified reviews were analyzed, and relevant data were extracted and recorded according to prior descriptions. Comparison of test accuracy data between established fall risk assessment measures and AFPS was performed descriptively.

## Results

### Test accuracy of the Aachen Fall Prevention Scale

#### Sensitivity and Specificity

Calculations were made based on 2 studies. The first sample retrieved from Knobe et al included 163 older adults (mean age 80.4 years, SD 6.4) [31]. The second one retrieved from Rasche et al contains 96 older adults with a mean age of 63.8 years (SD 7.02) [32]. Table 1 shows relevant data retrieved from the 2 studies.

Data from the study by Knobe et al [31] revealed a sensitivity of 56% (specificity of 64%) for the primary outcome parameter (self-assessment on 10-point Likert-scale) of the AFPS. If 1 out of the 3 outcome parameters of the AFPS is used to determine a positive result, then sensitivity could be increased up to 93%, whereas specificity thereby decreases to 11%. Calculations based on the data retrieved from the study by Rasche et al [32] showed a sensitivity of 66.7% (specificity 88.1%) for the primary outcome parameter of the AFPS. If just 1 out of the 3 parameters is used to determine a positive result, the sensitivity was again 66.7%, with a specificity of 84.5%.

**Figure 1:**
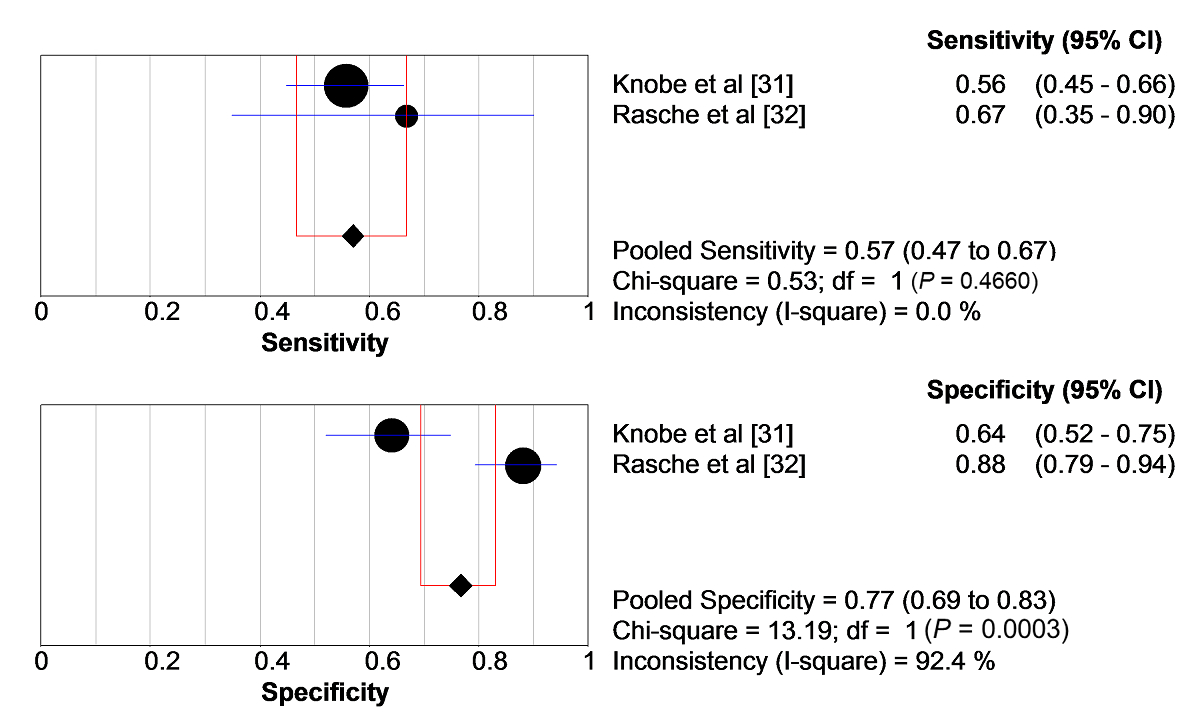
Pooled sensitivity and specificity regarding the primary outcome parameter of the Aachen Falls Prevention Scale. circles: study samples sensitivity/specificity; blue bars: CI of sensitivity/specificity; diamond: pooled sensitivity/specificity; red lines: CI of pooled sensitivity/specificity.

Regarding the primary outcome of the AFPS (third subscale; 10-point Likert-type scale), pooled sensitivity is 57.0% (95% CI 0.467-0.669) and specificity is 76.7% (95% CI 0.694-0.831; see Figure 1).

**Figure 2:**
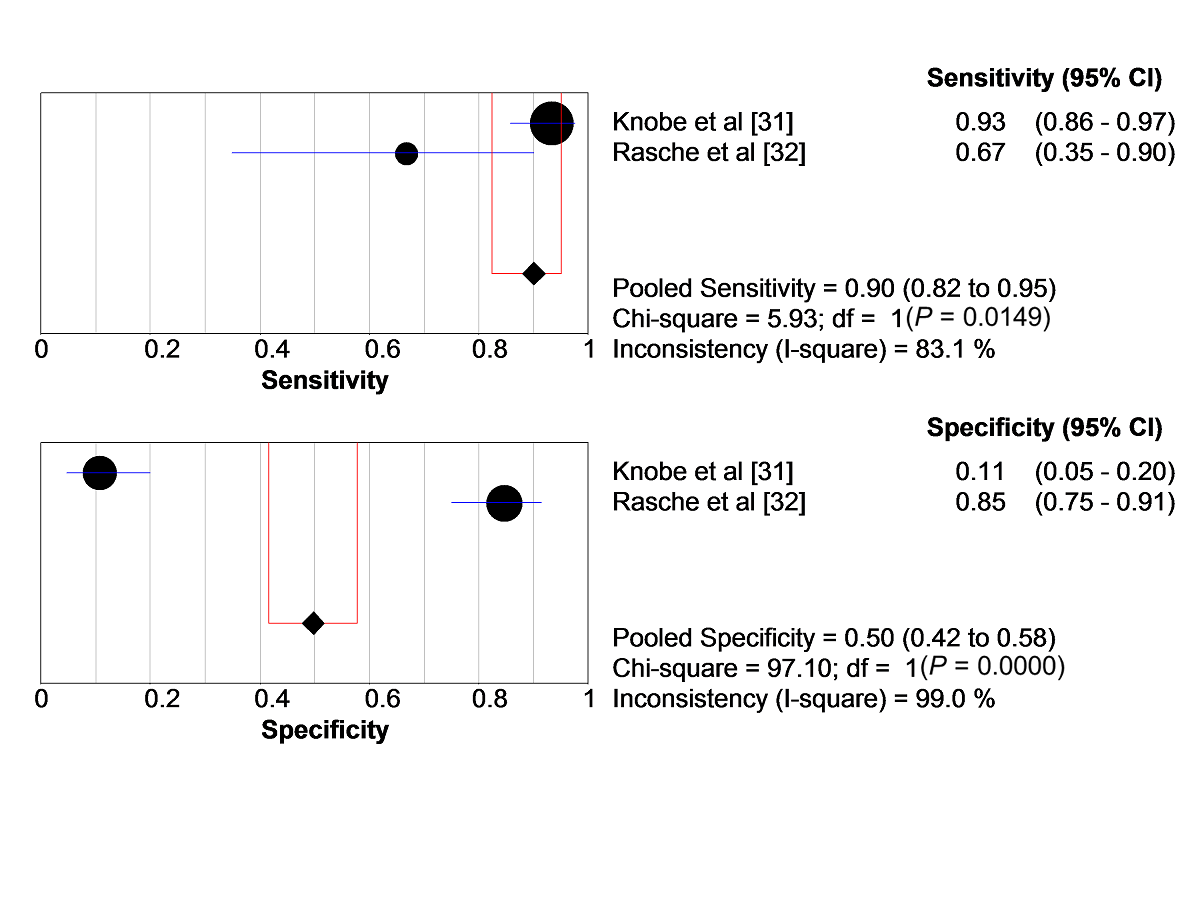
Pooled sensitivity and specificity regarding 1 out of the 3 steps of the Aachen Falls Prevention Scale. circles: study samples sensitivity/specificity; blue bars: CI of sensitivity/specificity; diamond: pooled sensitivity/specificity; red lines: CI of pooled sensitivity/specificity.

If 1 out of the 3 subscales of the AFPS is used to determine a fall risk, pooled sensitivity is increases up to 90% (95% CI 0.824-0.951) and specificity decreases to 50% (95% CI 0.42-0.58; see Figure 2).

#### Receiver Operating Curves and Area Under the Curve

**Figure 3:**
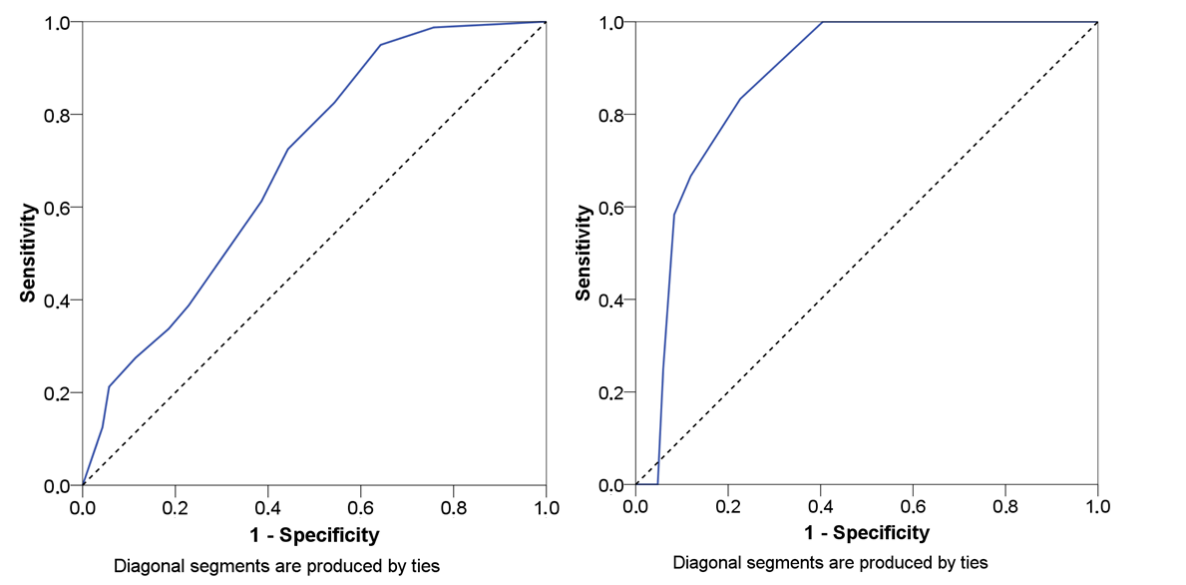
Receiver operating curves regarding primary outcome parameter (third subscale) of the Aachen Falls Prevention Scale to discriminate between fallers and nonfallers. Left side: Knobe et al [31] and right side: Rasche et al [32]; blue lines: receiver operating curves.

Following ROCs are described regarding the different outcome parameters of the AFPS. Calculations were made with SPSS. Figure 3 shows the test criteria of the primary outcome parameter of the AFPS using ROCs to discriminate between fallers and nonfallers. For the sample retrieved from the study by Knobe et al [31], the AUC for the primary outcome parameter of the AFPS was 0.692 (SE 0.043) and a 95% CI of 0.606-0.777. The AUC for the data retrieved from the study by Rasche et al [32] was 0.873 (SE 0.04) with a 95% CI of 0.796-0.980.

**Figure 4:**
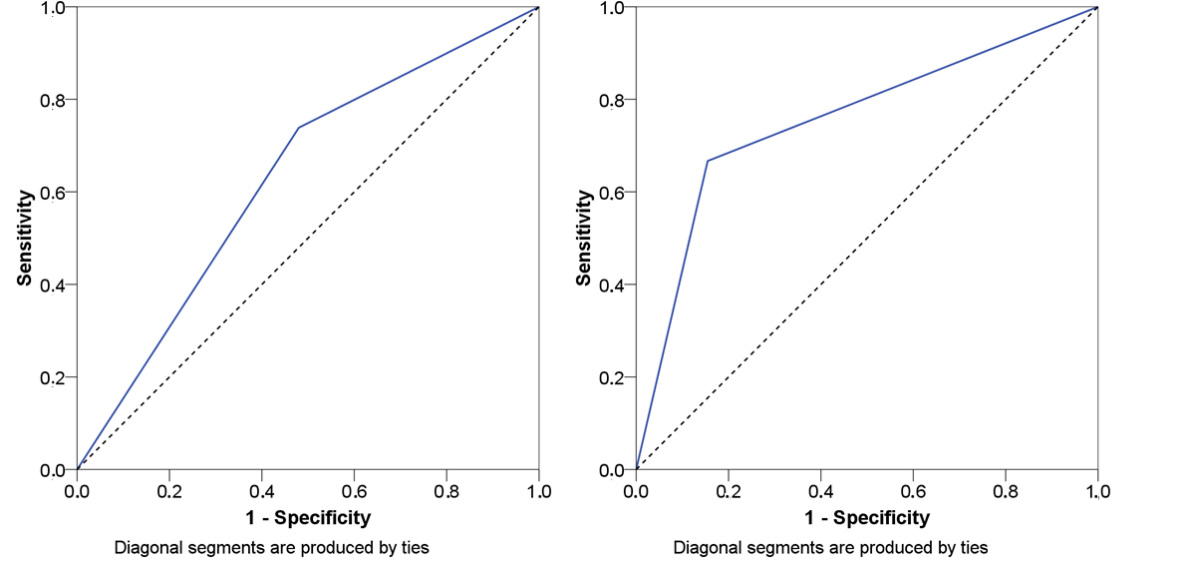
Receiver operating curves if 1 out of the 3 subscales of the Aachen Falls Prevention Scale is used to determine a fall risk. Left side: Knobe et al [31] and right side: Rasche et al [32]; blue lines: receiver operating curves.

Figure 4 shows the test criteria of ROCs to discriminate between fallers and nonfallers for the AFPS if 1 out of the 3 subscales is used to determine a fall risk.

The AUC for 1 out of the 3 subscales was calculated to 0.629 (SE 0.044) and a 95% CI ranging from 0.543 to 0.716 for the data extracted from the study by Knobe et al [31]. The sample retrieved from the study by Rasche et al [32] revealed an AUC of 0.756 (SE 0.084) and a 95% CI ranging from 0.592 to 0.920.

**Table 1:** Data extracted for calculating sensitivity and specificity of the Aachen Falls Prevention Scale.

Variable		Faller (score)		Nonfaller (score)	
		Knobe et al (2018) [31]	Rasche et al (2018) [32]	Knobe et al (2018) [31]	Rasche et al (2018) [32]
**Aachen Falls Prevention Scale (primary outcome)**					
	Subjective risk of falling ≥5^a^	49^b^	8^b^	27^c^	10^c^
	Subjective risk of falling <5	39^d^	4^d^	48^e^	74^e^
	Total	88	12	75	84
**Aachen Falls Prevention Scale (1 out of the 3 criteria)**					
	Balance test or Self-test ≥5 or subjective risk of falling ≥5^f^	82^b^	8^b^	67^c^	13^c^
	Balance test + or Self-test <5 or subjective risk of falling <5	6^d^	4^d^	8^e^	71^e^
	Total	88	12	75	84

^a^Main outcome of the AFPS was positive (>5 points in the subjective fall risk assessment).

^b^Correct positive.

^c^False positive.

^d^False negative.

^e^Correct negative.

^f^At least 1 of the 3 criteria of the AFPS was positive and compared with the number of fall incidents (n≥2, or n ≥1 + 1 injury) within the last year.

### Comparison of the AFPS with Established Assessment Measures

**Figure 5:**
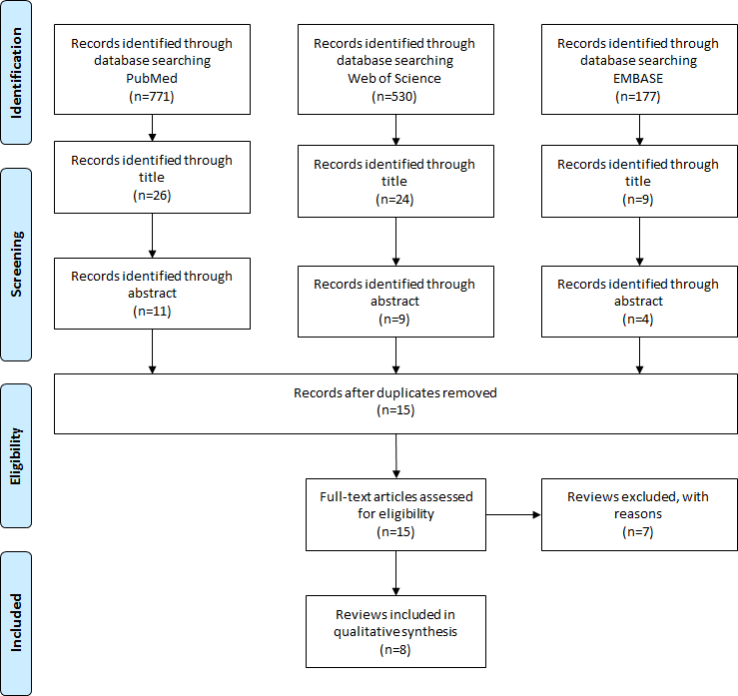
Results of the review of systematic reviews according to Preferred Reporting Items for Systematic Reviews and Meta-Analysis.

Figure 5 shows the article identification and selection process. In total, 948 articles were identified through keyword and reference search within PubMed and EMBASE databases. Of them, 937 articles were excluded after title and abstract screening. The remaining 11 articles were read full-text. Of them, 4 articles were excluded as these were unavailable to the authors. A further article was excluded as it did not fit the scope of this review. The remaining 6 articles were included in the review [25,26,36-41]. For all 6 reviews, the R-AMSTAR score was higher than 22 points (mean 40 points, range: 36-42 points).

#### Extracted Data

Table 2 gives an overview of the identified articles using the previously defined parameters: publication-related information, name of fall risk assessment measure, sample size, sensitivity, specificity, and AUC.

#### Data Aggregation and Comparison of Fall Risk Assessment Measures

Table 3 shows the extracted sensitivity and specificity values for the different fall risk assessment measures by means of mean value and range. Further corresponding values of the AFPS were included for comparison.

**Table 2:** Overview of identified reviews and extracted data for the meta-analysis.

Study and fall risk assessment measure		Sample size, N		Sensitivity, %		Specificity, %		Area under the curve (SE)	
**Perell (2001) [39]**									
	Berg Balance Scale		—^a^		77.0		86.0		—
	Dynamic Gait Index		133		85.0		38.0		—
	Elderly Fall Screening Test		361		93.0		78.0		—
	Timed Up and Go		30		87.0		87.0		—
	Performance-Oriented Mobility Assessment (Tinetti)		79		80.0		74.0		—
**Oliver et al (2004) [26]**									
	Downton Fall Risk Index		135		90.6		26.8		—
	Innes Score		2968		89.3		73.5		—
	Morse Score		2689; 483		73.2; 95.7		75.1; 54.0		—
	Schmid Score		2405		92.5		78.2		—
	STRATIFY^b^		395; 446; 432		93; 54.4; 73.7		87.7; 87.6; 45.2		—
**Aranda-Gallardo et al (2013) [36]**									
	Hendrich Fall Risk Model		—		62.8		64.0		—
	Morse Fall Scale		—		75.5		67.7		—
	STRATIFY		—		80.0		67.5		—
**Matarese et al (2014) [40]**									
	Hendrich II Fall Risk Model		—		92		37		—
	STRATIFY		—		63		71		—
**Lusardi et al (2017) [25]**									
	Berg Balance Scale		1130		41		88		—
	Five Times Sit-To-Stand test		3319		59		63		—
	Timed Up and Go (cut off >0.74 s/≥12 s)		6410		56; 31		65; 85		—
	Performance-Oriented Mobility Assessment (Tinetti)		1374		53		69		—
	Single-Limb stance (cut off >6.5/>12.7)		3015		19; 90		63; 49		—
**Park and Lee (2017) [41]**									
	Berg Balance Scale		1690		72		73		0.84 (0.02)
**Nunan et al (2018) [37]**									
	Downton Fall Risk Index		—		91		39		—
	Five Times Sit-To-Stand test		—		86		91		—
	Timed Up and Go		—		49		72		—
	Performance-Oriented Mobility Assessment (Tinetti)		—		64; 85		66; 56		—
	STRATIFY		—		50		76		—
**Park (2018) [38]**									
	Berg Balance Scale		570		73		90		0.97 (0.02)
	Downton Fall Risk Index		231		84		26		—
	Hendrich II Fall Risk Model		1754		76		60		0.75 (0.05)
	Mobility Interaction chart		286		53		73		—
	STRATIFY		2245		89		67		0.81 (0.30)
	Timed Up and Go		427		76		49		0.80 (0.04)
	Tinetti Balance scale		284		68		56		—

^a^Not applicable.

^b^STRATIFY: St Thomas’s Risk Assessment Tool in Falling Elderly.

**Table 3:** Range of sensitivity and specificity of identified fall risk assessment measures compared with the Aachen Falls Prevention Scale.

Type of fall risk assessment measure and name		Mean sensitivity, % (range)	Mean specificity, % (range)	Area under the curve, mean (range)
**Functional**				
	Berg Balance Scale	65.8 (41-77)	84.3 (73-90)	0.90 (0.84-0.97)
	Timed Up and Go	59.8 (31-87)	71.6 (49-87)	0.80
	Performance-Oriented Mobility Assessment (Tinetti)	70.5 (53-85)	66.3 (56-74)	—^a^
	Five Times Sit-To-Stand test	72.5 (59-86)	77 (63-91)	—
**Multifactorial**				
	Downton Fall Risk Index	88.5 (84-91)	30.6 (26-39)	—
	Morse Score	81.5 (73.2-95.7)	65.6 (54.0-75.1)	—
	STRATIFY^b^	71.9 (50-93)	71.7 (45.2-87.7)	0.81
	Hendrich II Fall Risk Model	84 (76-92)	48.5 (37-60)	0.75
	Aachen Falls Prevention Scale (primary outcome parameter)	57.0	76.7	0.724 (0.692-0.756)
	Aachen Falls Prevention Scale (1 out of the 3 outcome parameters)	90.0	50.0	0.693 (0.629-0.756)

^a^Not applicable.

^b^STRATIFY: St Thomas’s Risk Assessment Tool in Falling Elderly.

## Discussion

### Principal Findings

#### Sensitivity and Specificity of the Aachen Falls Prevention Scale

In this paper, the sensitivity and specificity of the AFPS were determined using a meta-analysis based on 2 independent studies. The results showed that by using the primary outcome parameter of the AFPS to discriminate between fallers and nonfallers, a pooled sensitivity of 57.0% and a pooled specificity of 76.7% can be achieved. If discrimination between fallers and nonfallers is based on a positive subscale (risk of falling present), the pooled sensitivity can be increased to 90.0%, whereas the pooled specificity thereby decreases to 50.0%. The AFPS, thus, exhibits an adequate combination of the necessary abilities that a patient-driven self-assessment tool should have. If all 3 outcomes are used, the fall risk is overestimated rather than underestimated, as sensitivity is about 90.0%. As a result, older adults may become more proactive and conduct a professional fall risk assessment at a clinic, even though it might just be a false alarm. Furthermore, the 2 studies investigated by Knobe et al [31] and by Rasche et al [32] showed that the AFPS can be used by users independently via a paper manual as part of a postal survey or via a digital manual as part of a Web-based survey. This indicates that, in addition to the specific test accuracy parameters, this instrument also fulfills the requirement of patient-driven fall risk assessment as stated in the Introduction. To what extent, however, this instrument has a positive effect on the work of family doctors within the guidelines of fall risk assessment of older adults remains unclear.

### Comparison of the Aachen Falls Prevention Scale With Established Fall Risk Assessment Measures

The systematic literature research conducted in the second step identified 9 different fall risk assessment measures, which were examined in at least two independent reviews. Identified reviews revealed a variety of reported sensitivity and specificity values. Within the group of functional fall risk assessment measures, Timed Up and Go [25,37-39], Berg Balance Scale [25,38,39,41], and Performance-Oriented Mobility Assessment (POMA) [25,37,39] were most frequently discussed and analyzed within identified literature. The multifactorial fall risk assessment measures St Thomas’s Risk Assessment Tool in Falling Elderly (STRATIFY) [26,36-38,40] and Downton Fall Risk Index [26,37,38] were most frequently investigated within identified reviews.

The lowest sensitivity in a single study, as well as on average, was identified for the Timed Up and Go test, followed by the mountain balance scale. It is noteworthy that the functional assessments show a lower sensitivity in comparison with the characteristic values of the multifactorial assessments. Functional assessments, on the other hand, have on average a higher specificity than multifactorial assessments. Compared with these instruments, the AFPS has the advantage that a high specificity of 76.6% or a high sensitivity of 90.0% can be achieved by selecting the considered outcome parameter (primary or 1 of the 3 subscales).

The AFPS, thus, offers an approach for mapping the advantages of both a highly sensitive and a highly specific test. Although the databases need to be strengthened by further studies, results show a promising approach. Compared with all the risk assessment measures examined in this review, the AFPS shows similar performance based on calculated sensitivity, specificity, and AUC. In addition, the AFPS has the advantage that it can be used by patients or caregivers themselves to monitor the risk of individual falls in the long term.

### Limitations

The limitations of this study and the studies presented here are two-fold. On the one hand, limitations are to be discussed with regard to the investigation of the test accuracy of the AFPS, and on the other hand, limitations are to be discussed with regard to the systematic literature research conducted.

The study to examine the test accuracy is limited by the study size, which is small compared with the examination of established fall risk assessment measures. A total number of 259 persons were examined. The data were collected in a controlled telephone study and in an anonymous Web-based survey in the second study. For a more comprehensive investigation of the test accuracy, a stronger focus on controlled patient groups from the clinical context should be included in further studies to achieve a more detailed patient segmentation with regard to the risk of falls. Furthermore, the inclusion of only 2 studies did not allow for an in-depth analysis using the MetaDiSc1.4 software. The inclusion of further studies is necessary for the analysis using ROCs by MetaDiSc1.4. Thus, corresponding curves were analyzed using SPSS.

Regarding the literature review and comparison, further limitations need to be considered in the interpretation of the stated results. Correct data aggregation based on different identified reviews was challenging as different cut-off points were chosen but not reported comprehensibly. Furthermore, sensitivity and specificity values of established fall risk assessment measures are not drawn from results of primary studies but from reviews that have synthesized the results already. In this respect, the validity of the comparison must be limited. Furthermore, the parameters for the sensitivity and specificity of the individual fall risk assessment measures extracted from the reviews are based on a different number of studies and contain study populations of different sizes. Thus, the limitations of the identified reviews with regard to the significance of the parameters specified there are also relevant for this contribution. Given these limitations, the results should be interpreted with some caution, and further studies designed to investigate test accuracy by direct comparison with the same study population should be conducted.

### Conclusions

This study investigated the test accuracy of the AFPS as a patient-driven self-assessment tool compared with established tools such as Timed Up and Go, POMA, STRATIFY, or Downton Fall Risk Index. This study showed that the AFPS is a promising tool for patient-driven fall risk assessment. It is quick and easy to use.

The AFPS showed suitable pooled sensitivity (57.0%; 95% CI 0.467-0.669) as well as a suitable specificity (76.7%; 95% CI 0.694-0.831) regarding discrimination between fallers and nonfallers by primary outcome. Sensitivity of the AFPS could be increased up to 90.0% (95% CI 0.824-0.951) and a specificity of 50.0% (95% CI 0.420-0.580) if 1 out of the 3 parameters of the AFPS is used to discriminate between fallers and nonfallers.

Thereby, the AFPS shows an adequate combination of the necessary abilities that a patient-driven self-assessment tool should have. If it is used as prescribed (all 3 subscales are used), the fall risk is rather overestimated than underestimated. Thereby, older adults might get sensitized and consult a physician for clinical fall risk assessment even in the case of a false alarm.

The systematic analysis of existing reviews of fall risk measures shows the multitude of available measures and the range of associated sensitivity and specificity values. No outstanding measure was identified in this study, which illustrates the difficulty of selecting these measures in a clinical context. Nevertheless, we were also able to show that the newly developed AFPS is a suitable instrument with which fall patients and elderly people can independently assess and monitor their individual fall risk in the long term. In particular, the approach of bringing this method to the smartphone of affected or interested older adults using an app constitutes a promising approach, as its sensitivity and specificity are comparable with established fall risk assessment measures.

Nevertheless, the multitude of methods reviewed in this study was developed with a focus on clinical use, as the methods are intended to support the assessment of the risk of falls by physicians or medical specialists. Instruments that are supposed to start one step sooner in the process and enable the patient to assess the individual risk of falling independently are not yet widespread. One instrument that can be used in this context is the AFPS. Other studies have already shown that this instrument, in form of a health app, can be and is used by older adults to assess their individual risk of falling [24].

All in all, according to the investigated data, the AFPS and thus the AFPA are suitable approaches for increasing patient autonomy and simplifying the process of individual fall risk assessment. Through the application of AFPS and the further spread of AFPA, older people can be made aware of the risk of falling and clinical resources can be saved through the initial self-assessment by the older adults themselves.

## Abbreviations

AFPA: Aachen Falls Prevention App
AFPS: Aachen Falls Prevention Scale
AUC: area under the curve
POMA: Performance-Oriented Mobility Assessment
PRISMA: Preferred Reporting Items for Systematic Reviews and Meta-Analysis
R-AMSTAR: Revised Assessment of Multiple SysTemAtic Reviews
ROC: receiver operating curve
STRATIFY: St Thomas’s Risk Assessment Tool in Falling Elderly
NICE: National Institute for Health and Care Excellence
US PSTF: United States Preventive Service Task Force
